# Haematorrhacis or Meningeal Apoplexia

**Published:** 1884-04

**Authors:** 


### CASE DEPARTMENT.

### I.—HAEMATORRHACIS OR MENINGEAL APOPLEXIA.

### Apoplexia Spinalis in the Horse.

This occurrence must be very rare, for I find no reference to it in Veterinary literature, yet from personal experience it seems to me that *it can occur* more frequently than has been observed, as the construction of the vertebral column in the horse, and the exertions to which work horses are put, essentially favor it.

*Case* 1.—On Tuesday, October 14th, 1879, I was called in to see a horse belonging to a large pork-packing house in the vicinity of Boston.

The horse went to its work in an apparently well condition on the morning of the day in question, though it had previously had four days' rest; its food had been cut short however. It went into the city, a distance of three miles, with a load and returned apparently well. It then returned with another load with which it had to stop on an incline toward a draw-bridge; the exertions required to again start the load were very severe indeed. The horse stopped, its movements became irregular, *no profuse perspiration, however*, but seemed to lose control of its posterior extremities; its ankles knuckled under, and it finally fell and was brought home on a dray.

I saw it at 5 P. M., on the same day.

*Status praesens.*—Extremities cold, particularly the posterior, heart beats in general regular, with an occasional systolic intermittance. *Arterial* pulse weak and intermittant; dicrotic.

On endeavoring to make the patient move it was almost impossible to get him to stir from his position; its legs seemed like iron bars, not to be moved; it gave way in the ankles, knees and hocks, and we were fortunate in saving it from tumbling down.

Sensorium free; appetite voracious; *no fever;* urine clear and contained no albumen—the patient was insensible to pin prick posterior to the shoulders, but not on neck and head.

*Diagnosis.*—Paralysis of motor centres of spine from some unknown cause.

Ordered patient to be kept in slings.

*October* 15.—Patient stood immovable in the same place it had been left the night before.

Temperature 38.3° C. Pulse 60—irregular. Resp. 20. Condition same as before.

*October* 16.—Condition same; though patient is stronger, but still knuckles in posterior ankles if moved. *Urine clear and normal.*

Temp. 37.8° C. Resp. 18.

Ordered strychnine hypodermatically.

*October* 20.—Patient slowly improving; respiration, pulse and temperature normal, but still knuckles if moved.

To make a long story short, the patient steadily improved, and after four months' rest entirely recovered and is at work to this day, never having been ill since.

*Case II.*—On Sunday, November 7, was called to see an aged bay horse belonging to a retail coal-dealer. Patient was said to have been ill about a week; on Friday of the previous week the horse was loaded with a very heavy load of coal; the driver stopped him to rest on the brink of a very abrupt hill; started him; he strained violently, gave it up, and "*was gone in his legs*," especially the ankles. With great care and men to support it, was got back to the stable. A practitioner was called in who called it "indigestive colic;" he left some powders for the patient, saying "it would be all right the next day." That day never came!

On the Sunday that I was called in, I found that the horse had fallen early in the morning, and had not been able to get up; the owner said he had scarcely moved in his tracks for a week; there was no reaction in the posterior parts, even though I punctured through the skin with a pen knife. Its appetite had been good all the time. This morning the patient had thrown himself or fallen down with great violence. *The sensorium was free,* and though prostrate the animal would eat and drink as naturally as ever. Pulse accelerated, though strong and regular. Respirations as normal as they could be under the circumstances. Ears warm; lower limbs cold; *urine normal.*

A veterinary surgeon that was accidentally with me pronounced it "Spinal Meningitis."

*Diagnosis.—Spinal paralysis due to unknown causes.*

On Monday, the 8th of November, chloroformed the patient at the request of the owner; though it still eat and drank, it could not get up either forward or behind; it could raise its head, however, and was thoroughly conscious of all surroundings.

Tuesday, November 9th, autopsy, which required five hours to complete.

All internal organs comparatively normal. Some chronic interstitial and capsular hepartites. Stomach normal and full of food. Brain normal. A slight increase of the sub-arachnoidal fluid in spinal cord. The latter presented a normal appearance from the brain to the brachial enlargement. Here we found an extensive *extra meningeal hemorrhage of recent date, composed of clotted blood, surrounding one that had taken place earlier and was harder and of greater consistency, and somewhat paled out. The spinal cord was compressed like a full hose pressed together at a given point with the fingers. Both of the blood sinuses of the spinal canal ruptured at this point.*

CONCLUSIONS.

This case occurring so soon after the first, gave me the "key" by which to form an eventually favorable prognosis with reference to it, and as reported, the patient recovered.

The anatomical conditions; that is, the movability of the first dorsal and last cervical veterbrae in the spine of the horse, the fact that this part of the neck forms the *fulcrum,* the legs being the lever by which a horse starts a heavy load, the extension which it gives to its neck at such a moment, can in given cases give occasion to such an accident. I could not discover any degeneration of the walls of the sinuses. I am sure I saw just such a case while a student at Berlin in a balky horse, that gave way suddenly when finally "buckling to its work." The autopsy, which did not extend beyond the brain, revealed absolutely nothing.

Recovery takes place by the gradual absorption of the transudated blood, the cord finally assuming the normal proportion and condition.

Erb says (Ziemssen's Handbook d. Pathologie—Ruckenmark-krankheiten) "under the name Haematorrhacis is comprehended all hemorrhages which take place in, around, and between the meninges of the spinal cord. They are very rare occurrences, but are accompanied by quite characteristic phenomena."

I will state that I sent the spinal cord, in toto, to the Pathological Department of Harvard Medical School, and was informed that they had never met with such a case in man. "Nothing is known of predisposing causes in man, such as fatty or artheromatous degeneration." As accidental causes, traumata play the chief part. Inflammatory and carious processes in the vertebra have occasionally given occasion to meningeal hemorrhages in man. Severe bodily exertions may occasion them. Rupture of aneurisms in the sinuses have been reported."

"Hemorrhages between the dura and canal (that is from the sinuses), are the most frequent variety. They must be very extensive when they cause compression of the cord and interfere with its functions."

"The trouble presents itself suddenly; the patient appears as if struck with apoplexy, but *almost always without any disturbance of the cerebral functions. Prodromal indications are very seldom observed.*"

"The veterbral column is stiff and painful, paralytic phenomena soon appear; movements are impossible; posterior to the lesion a more or less extensive degree of anesthesia develops."

"The phenomena remain apparently stationary for a time, but improvement gradually takes place. The prognosis is to be pronounced favorable in most cases. In the course of several weeks or months, recovery generally results. On the other hand some cases end fatally."

"The diagnosis is often bound with many difficulties." B.

